# Evolutionary Trends, Regional Differences and Influencing Factors of the Green Efficiency of Agricultural Water Use in China Based on WF-GTWR Model

**DOI:** 10.3390/ijerph20031946

**Published:** 2023-01-20

**Authors:** Ruifan Xu, Jianzhong Gao

**Affiliations:** College of Economics and Management, Northwest A & F University, Yangling 712100, China

**Keywords:** green efficiency, agricultural water use, evolution trend, regional differences, influencing factors

## Abstract

Improving the green efficiency of agricultural water use is a key way to promote the sustainable utilization of agricultural water resources and sustainable development of economy and society. This work calculated and analyzed the evolution trend, regional differences and driving factors of the green efficiency of agricultural water use in China from the perspective of the water footprint. The results show that the green efficiency of agricultural water use in China shows a fluctuation trend of first declining and then rising from 1997 to 2020, after which the average efficiency dropped from 0.538 in 1997 to 0.406 in 2009, and then rose rapidly to 0.989 in 2020, with an average annual growth rate of about 3.6%. From a regional perspective, the green efficiency of agricultural water use in the eastern region was the highest (0.594), above the national average (0.538), followed by the western region (0.522), with the central region in last (0.491), with significant regional differences. The spatial differences in the green efficiency of available agricultural water in China shows a fluctuating downward trend. The Gini coefficient fluctuated from 0.271 in 1997 to 0.182 in 2020, with an average annual growth rate of about −1.4%. The main source of this regional difference was super-variable density, followed by the difference between the eastern and the central regions. The influence of urbanization level, water-saving level and agricultural trade on the green efficiency of agricultural water use was always positive and the influence of industrialization level was always negative; among them, the urbanization level, water-saving level and industrialization level had a greater impact on Northeast China, and agricultural trade had a greater impact on Southeast China. Therefore, this work puts forward relevant policy recommendations.

## 1. Introduction

As a basic natural resource and a strategic economic resource, water has been vital to human survival, economic growth and social development [1]. With global climate change, rapid population growth and industrial restructuring, agricultural water use continues to decrease, posing serious challenges to food production [2]. By 2030, global food demand will increase by 35.0%, energy demand will increase by 50.0%, existing water resources will not be able to meet the needs of a growing population and 3.2 billion people will face water shortages [3]. In addition, modern agricultural production has caused a large amount of non-point source pollution, posing a serious threat to the green development of agriculture; the largest source of water pollution is agriculture [2]. China feeds 21.0% of the world’s population with 6.5% of its water resources. However, the water resources per capita are very poor: less than a quarter of the world average [4]. The total water use in China was 581.3 billion m^3^ in 2020, and agricultural water accounted for 62.1%. In addition, giving priority to ensuring food security and developing the agricultural economy has not only seriously restricted water use and caused water shortages, but also directly threatens ecological security [5]. According to the Second National Pollution Sources Census Bulletin, agricultural non-point source pollution was more serious in 2017: the COD and NH3-N emissions were 10.671 and 0.216 million tons, accounting for 49.8% and 22.4% of the total national emissions, respectively. Therefore, how to reduce resource inputs and environment outputs and realize efficient and green use of water resources is the key to solving the problem of water resources and food security.

Water utilization efficiency is a broad concept including engineering and production concepts [6]. From the perspective of engineering, improving water efficiency could be achieved by reducing the water loss, such as with channel seepage prevention and drip irrigation [7]. From the perspective of production, water efficiency is defined as the physical and economic output of water use, representing the economic benefits or production generated by each 1 m^3^ water resources [8]. Currently, the effectiveness of water resource exploitation is considered from three perspectives; irrigation water [9,10], rain-fed sourcing [11,12], and other water inflows [13]. Irrigation water was originally regarded as essential to agricultural water resource management and irrigation efficiency (IE) was defined as the ratio of water consumed by the crop to the amount of field water supplied through surface flow, sprinklers or drip irrigation [14]. However, in an agricultural production system without irrigation equipment, rainwater is very important for agricultural production, because it is the only water source available for crops [10]. The rainwater use rate (RUR), which is calculated as the proportion of crop rain water evapotranspiration (ET) relative to total rainfall, represents the efficiency of precipitation use estimated from the perspective of rain sourcing [11]. The generalized efficiency index (GE) refers to the ratio of field crop ET to the amount of total water inflow; it was established to determine the use of both irrigation water and rain water [13]. Evaluating water use efficiency by treating blue and green water resources equally in any context, including irrigation and rain sources, are advantages of this indicator, since green water accounts for the vast majority of water consumed in croplands [15].

With the transformation of crop water analysis paradigms and the environmental pollution caused by water resource utilization becoming increasingly prominent, the concept of a water footprint (WF) was introduced by Hoekstra (2003) in the early 2000s [16]. Crop WF (CWF) refers to the volume of fresh water that is consumed during the crop growing period, and it generally consists of green, blue and grey water footprints [17]. That it measures the impact of crop production on the consumption and pollution of all water resources simultaneously constitutes an advantage of CWF for the quantification of water resources, and in this way, it differs from the three perspectives mentioned above [18]. As the largest water sector in China, agriculture suffers from problems such as water shortage, water pollution and low efficiency, which seriously restricts the high-quality development of agriculture. Reducing WF and improving the green efficiency of agricultural water use are the ultimate goals of CWF assessment [19]. Therefore, CWF accounting constitutes the basic premise for the evaluation of water use efficiency, agricultural water resource savings and conservation research, and agricultural water green efficiency research is emerging gradually [20]. Wang et al. (2014) and Xu et al. (2019) combined WF with the water productivity (WP) index to measure and analyze water use efficiency for food production from the perspectives of water volume and system stress [21,22]. Cao et al. (2021) examined water resource efficiency (WRE) and the effects of effective crop water use on major food crops (rice, wheat, maize, beans and tubers) from the perspective of the water footprint [23]. Zhang et al. (2022) developed a water-footprint-based interval fuzzy robust fractional programming (WF-IFRFP) model through integrating water footprint (WF), interval parameter programming (IPP) and fuzzy robust optimization (FRP) into fractional programming (FP) and applied this to the Hetao Irrigation District to improve the green efficiency of local water resources and optimize the planting structure of wheat, corn and sunflower [24]. Cao et al. (2020) combined the water footprint and agricultural water use paradigms to construct a hybrid framework for agricultural water utilization and efficiency evaluation and conducted an empirical study on the framework by taking the major cereals (wheat, maize and rice) in 31 provinces, autonomous regions and municipalities of China as examples [25]. The above content enriched the related research on CWF and the green efficiency of agricultural water use. However, the above research mainly focused on the green efficiency of agricultural water use in the narrow sense (water use efficiency of grain production). Agriculture, as an important industrial sector in China’s national economy, includes planting, forestry, animal husbandry and fishery; ignoring the water use efficiency in the production process of forestry, animal husbandry and fishery products will bring some deviations to the comprehensive evaluation of the board agricultural water use green efficiency. In addition, the above studies only regarded water resources as input factors, ignoring the input of other factors such as land, labor, machinery and energy, and the unexpected output only considered water environmental pollution, ignoring agricultural carbon emissions, which is contrary to the coordinated development of economic and social resources and environmental protection in the concept of sustainable development.

China is a vast country; there are great differences among different regions in resource endowment, social and economic development, agricultural science and technology level. Agricultural water use has temporal and spatial heterogeneity. Therefore, exploring the spatio-temporal characteristics of agricultural water efficiency has become a necessary means to coordinate regional agricultural water distribution. Based on this, many scholars have used exploratory spatial data analysis [6,26], kernel density analysis [27], spatial convergence models [20] and social network analysis [28] to analyze the spatial and temporal characteristics and reveal significant regional heterogeneity. However, the two-dimensional kernel density analysis can only reveal the spatial and temporal variation characteristics in a specific year, and missing years with outliers can skew the results. Meanwhile, three-dimensional kernel density could depict the spatial and temporal characteristics in all years, improving the accuracy of the analysis results. In addition, it was difficult to effectively eliminate the regional differences in green efficiency of agricultural water use in China. The above methods could not effectively explain the underlying causes of regional differences and answer questions such as “How big is the regional difference”, “Where is the overall difference mainly coming from” and “What kind of dynamic change characteristics appear with the temporal variation”, etc., which have important practical significance and reference value for scientifically predicting and improving the green efficiency of agricultural water use in all regions.

Breaking through the shackles of resources and the environment on agricultural production and solving the contradiction between agricultural production and environmental protection is the fundamental requirement to achieve a green, ecological, intensive and efficient sustainable development pattern. Therefore, which factors will affect the realization of the green development of agricultural water use? Most studies used Tobit [29], OLS (ordinary least squares) [30], GMM (Gaussian mixture model) [31] and LMDI (logarithmic mean Divisia index) [32] models to explore the influencing factors, mainly from the aspects of water resource endowment [2], economic development level [33], government control [34], population [35], technical level [36], industrial structure [37] and environmental regulation [38]. These studies belong to static model analysis, which separates the influencing factors from the change results statically, ignoring the dynamic analysis between the spatio-temporal pattern of green efficiency of agricultural water use and its influencing factors in different regions, and it is difficult to explain the differences in the influence of different influencing factors on green efficiency in different time and regions.

The possible marginal contributions of this manuscript are as follows: (1) From the perspective of water footprint, blue water and green water consumed in the production process of planting, forestry, animal husbandry and fishery products, as well as water pollution (grey water) and environmental pollution (agricultural carbon emissions) were included in the input–output analysis framework, aiming to break through the previous problem of focusing only on the water efficiency of grain production and ignoring agricultural carbon emissions and comprehensively evaluate the broad agricultural water use green efficiency of each region. (2) The dynamic evolution of the green efficiency of agricultural water use in each region was investigated by drawing the three-dimensional kernel density. (3) Considering the spatial and temporal heterogeneity of the regions, a geographically and temporally weighted regression (GTWR) model was used to explore the influencing factors in green efficiency of agricultural water use. The research ideas of this paper are as follows: (1) From the perspective of water footprint, a broad agricultural water use green efficiency evaluation index system was comprehensively constructed, including planting, forestry, animal husbandry and fishery. (2) The Super-SBM, kernel density estimation model and Dagum Gini coefficient were used to measure and reveal the evolution trend and regional differences in green efficiency of agricultural water use in China. (3) The GTWR model was used to analyze the influencing factors in green efficiency of agricultural water use, in order to provide a scientific decision making basis for improving the green efficiency of agricultural water use in China, realizing sustainable utilization of agricultural water resources and ensuring national food security.

## 2. Materials and Methods

### 2.1. Indicator Selection and Data Source

#### 2.1.1. Index System for Measuring Green Efficiency of Agricultural Water Use

As shown in Table 1, the measurement of green efficiency of agricultural water use mainly involves the selection of input variables and output variables, among which the input variables include land, labor, agricultural capital, agricultural water resources and technology. Output variables were divided into expected output and unexpected output. Among them, expected output included the total output value of the primary industry (converted to 1997 at constant prices) and total grain output of each province (municipality, district) in China, while unexpected output included agricultural grey water footprint and agricultural carbon emissions.

#### 2.1.2. Index System of Influencing Factors of Green Efficiency of Agricultural Water Use

The factors affecting the agricultural water use green efficiency in China are complex and complicated. In this paper, based on the availability of data, nine indicators were selected from five aspects—natural factors, socio-economic factors, policy factors, scientific and technological factors, environmental factors and external opening factors—to analyze their influence on agricultural water use green efficiency in China and explore the spatial and temporal differentiation of the influencing factors. The specific influencing factors are shown in Table 2.

Based on the principle of index independence, this paper uses the variance inflation factor (VIF) method to test for the multicollinearity test (Table 3). (According to the variance inflation factor (VIF) method, the larger the VIF, the more seriously the multicollinearity is indicated. Generally, if 0 < VIF < 10, it indicates that there is no multicollinearity among the indicators; if VIF ≥ 10, it indicates that there is strong multicollinearity among the indicators.) After the test, the average VIF value of each influencing factor was 1.95, and the maximum VIF value was 2.89, which is much less than 10; therefore, there is no multicollinearity among the indicators.

#### 2.1.3. Data Sources and Description

**Figure 1:**
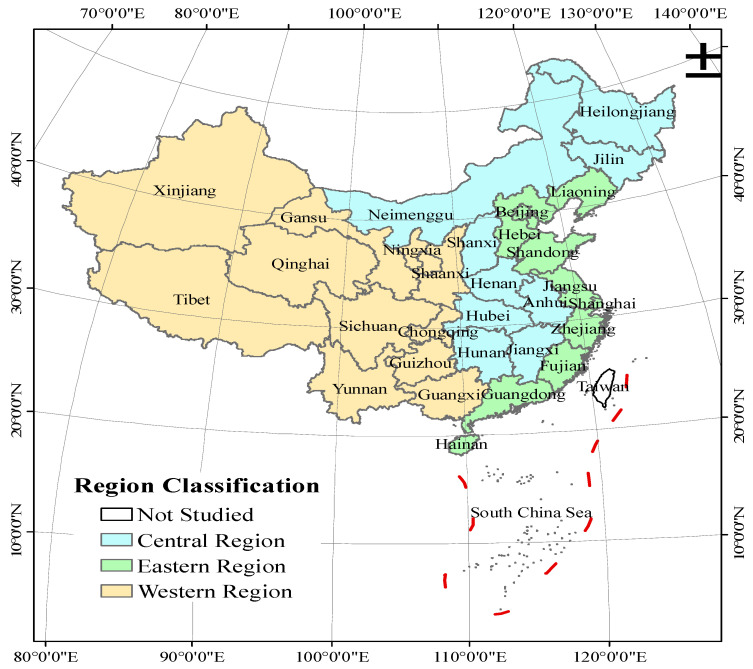
The study area overview map.

Considering the availability of data, this paper selected 31 provinces, municipalities and districts in China (except Hong Kong, Macao and Taiwan) as the study area, and the time range was from 1997 to 2020 (Figure 1). The data were obtained from the China Statistical Yearbook, China Agricultural Yearbook, China Rural Statistical Yearbook, Compilation of Statistical Data in the 60 Years of New China, China Environmental Statistical Yearbook, statistical yearbooks of various provinces and cities, etc. The data of some missing years were made up via interpolation. Data such as livestock and poultry feeding cycle, daily excretion and urine volume, pollutant content per unit of feces and urine, and pollutant loss rate per unit of feces and urine were referenced from “Technical Report on the Survey of Pollution in China’s Large-Scale Livestock and Poultry Farming Industry”.

### 2.2. Research Method

#### 2.2.1. Water Footprint Model

Agricultural water footprint indicates the total freshwater resources consumed by a country, region or individual consuming all agricultural products and services in a certain period of time, and it is the sum of blue water, green water and grey water footprints [50]. Among them, the blue water footprint refers to the total amount of surface water and groundwater consumed during the growth of crops, the green water footprint refers to the effective amount of precipitation absorbed and utilized by crops while growing, and the grey water footprint refers to the amount of freshwater resources required to dilute the environmental pollutants produced by fertilizer and pesticide applications to the standard concentration of water quality; please refer to Appendix A for the specific calculation formula.

#### 2.2.2. Super-SBM Model

The Super-SBM model was first proposed by Tone in 2002, and combines the advantages of both the super-efficiency DEA model and the SBM model to exclude multiple efficiency values of 1 at the same time from the production possibility set, thus allowing further analysis of efficiency differences between decision units [51,52]. In addition, it can also evaluate the environmental efficiency under the condition of undesired output, which is often generated simultaneously in the process of agricultural development. Therefore, the Super-SBM model is used in this paper to evaluate the agricultural water use green efficiency of China. Suppose there are *n* decision-making units in the system to be evaluated, and each unit contains input, expected output $S_{1}$ and non-expected output $S_{2}\;$3 vectors, which are, respectively: $x\in R^{m}$, ${\;y}^{g}\in R^{S_{1}}$, ${\;y}^{b}\in R^{S_{2}}$. Meanwhile, the matrices X, $\;Y^{g}$, $\;Y^{b}\;$are defined as follows:

$$\left[X\right]={\left[x_{1},\;\cdots ,\;x_{n}\right]}^{T}\in R^{m\times n},\;\left[Y^{g}\right]={\left[y_{1}^{g},\;\cdots ,\;y_{n}^{g}\right]}^{T}\in R^{s_{1}\times n},\;\left[Y^{b}\right]={\left[y_{1}^{b},\;\cdots ,\;y_{n}^{b}\right]}^{T}\in R^{s_{2}\times n}$$

X>0*,*$\;Y^{g}>0$*,*$\;Y^{b}>0$, the production possibility set *P* is defined as:

$$P=\left\{\left(x,\;y^{g},\;y^{b}\right)|x\geq x,\;y^{g}\geq Y^{g},\;y^{b}\geq Y^{b},\;\geq 0\right\}$$

Then, the SBM model based on variable return to scale can be expressed as:

(1) $$P^{*}=min\frac{1-\frac{1}{N}\sum_{n=1}^{N}S_{n}^{x}/x_{k_{n}^{\prime }}^{t^{\prime }}}{1+\frac{1}{M+I}\left[\sum_{m=1}^{M}S_{m}^{y}/y_{k_{m}^{\prime }}^{t^{\prime }}+\sum_{i=1}^{I}S_{i}^{b}/b_{k_{i}^{\prime }}^{t^{\prime }}\right]}$$

s.t.

$$\;x_{k_{n}^{\prime }}^{t^{\prime }}=\overset{T}{\underset{t=1}{\sum }}\overset{K}{\underset{k=1}{\sum }}\lambda_{k}^{t}x_{kn}^{t}+S_{n}^{x},\;n=1,\;\cdots ,\;N$$

$$y_{k_{m}^{\prime }}^{t^{\prime }}=\overset{T}{\underset{t=1}{\sum }}\overset{K}{\underset{k=1}{\sum }}\lambda_{k}^{t}y_{km}^{t}-S_{m}^{y},\;m=1,\;\cdots ,\;M$$

$$b_{k_{i}^{\prime }}^{t^{\prime }}=\overset{T}{\underset{t=1}{\sum }}\overset{K}{\underset{k=1}{\sum }}\lambda_{k}^{t}b_{ki}^{t}+S_{i}^{b},\;i=1,\;\cdots ,\;I$$

$$\lambda_{k}^{t}\geq 0,\;S_{n}^{x}\geq 0,\;S_{m}^{y}\geq 0,\;S_{i}^{b}\geq 0,\;k=1,\;\cdots ,\;K$$

In Equation (1), $P^{*}$ represents the agricultural water use green efficiency; *N*, *M* and *I* are input, expected output and non-expected output indicators, respectively; *x*, *y* and b are vectors of three indicators, respectively; and $S_{n}^{x},\;S_{m}^{y},\;S_{i}^{b}\;$ are the relaxations of the three indices, respectively. $x_{k_{n}^{\prime }}^{t^{\prime }},\;y_{k_{m}^{\prime }}^{t^{\prime }},\;b_{k_{i}^{\prime }}^{t^{\prime }}\;$ represent the production units of $k^{\prime }\;$in $t^{\prime }$ period input–output value; $\lambda_{k}^{t}$ is the weight coefficient of the decision-making unit (DMU). When $P^{*}$ ≥ 1, it indicates that the DMU is at a high efficiency level. When $P^{*}$ < 1, it means that there is a certain efficiency loss, and the agricultural water use green efficiency can be improved by optimizing the input amount, expected output and unexpected output.

#### 2.2.3. Dagum Gini Coefficient

The Gini coefficient and subgroup decomposition method proposed by Dagum (1997) [53] were used to analyze the differences and sources of agricultural water use green efficiency in China. In this paper, the overall difference in green efficiency of agricultural water use in China was divided into three parts: intra-group difference, inter-group difference and super-variable density. The intra-group difference referred to the internal difference of eastern, central and western regions, and the inter-group difference referred to the difference between central-western, eastern-western and eastern-central regions. The super-variable density reflected the contribution of overlapping between different regions to the overall gap. The calculation formula is as follows:

(2) $$G=\frac{\sum_{j=1}^{k}\sum_{h=1}^{k}\sum_{i=1}^{n_{j}}\sum_{r=1}^{n_{h}}\left|y_{ji}-y_{hr}\right|}{2n^{2}\bar{y}}\;$$

In Equation (2), G is the global population Gini coefficient, which can reflect the global difference of agricultural water use green efficiency in China and be decomposed into intra-group difference ($G_{w}$), inter-group difference ($G_{nb}$) and super-variable density ($G_{t}$). The calculation method is similar to that for the global population Gini coefficient. K is the number of regions; j and h are the region numbers; $n_{j}$ and $n_{h}$ are the number of provinces (municipalities, districts) in the region j and h, respectively; *i* and *r* are the number of provinces (municipalities, districts) in the region *j* and *h*, respectively; and $y_{ji}$ is the agricultural water use green efficiency in province (municipalities, districts) i of region j. $y_{hr}$ is the agricultural water use green efficiency in province (municipalities, districts) r of region h.

#### 2.2.4. GTWR Model

The traditional geographically weighted regression model only considers the spatial dimension, which solves the problem of spatial non-stationarity but ignores the temporal effect. The GTWR model proposed by Huang et al. (2010) [54] incorporates the temporal factor on the basis of the spatial-only geographically weighted regression model, analyzes the variables from a three-dimensional perspective, and identifies the non-stationarity of both temporal and spatial components simultaneously. It has been widely used in various aspects of geography. The geographically and temporally weighted regression is as follows:

(3) $$y_{i}=\beta_{0}\left(u_{i},\;v_{i},\;t_{i}\right)+\underset{k=1}{\sum }\beta_{k}\left(u_{i},\;v_{i},\;t_{i}\right)x_{ik}+\varepsilon_{i},\;i=1,\;2,\;\cdots ,\;n$$

In Equation (3), $y_{i}$ is the agricultural water use green efficiency and $x_{ik}$ is the *k*th influencing factor of the ith sample. $\left(u_{i},\;v_{i},\;t_{i}\right)$ represents the space–time coordinates of the ith sample, and $t_{i}$ is the time distance. $\varepsilon_{i}$ is the random disturbance term; $\beta_{k}\left(u_{i},\;v_{i},\;t_{i}\right)$ represents the regression coefficient of the *k*th influencing factor at the regression point *i*. The local linear estimation of the geographically and temporally weighted regression model can be used to obtain the estimated value of each regression coefficient at the sample point *i*. The estimation method is as follows:

(4) $$\hat{\beta }\left(u_{i},\;v_{i},\;t_{i}\right)={\left[X^{T}W\left(u_{i},\;v_{i},\;t_{i}\right)X\right]}^{-1}X^{T}W\left(u_{i},\;v_{i},\;t_{i}\right)Y$$

In Equation (4), the weight $W\left(u_{i},\;v_{i},\;t_{i}\right)$ is the distance function between sample point i and other sample points, which adopts the Gaussian distance function. The closer the distance between sample point *i* and other neighboring points, the higher the weight.

Therefore, this paper used the GTWR model to explore the influencing factors and spatial–temporal heterogeneity of the green efficiency of agricultural water use in China from 1997 to 2020 to realize the deconstruction of local parameter effects, then analyze the spatial and temporal heterogeneity patterns of the magnitude of the effects of each influencing factor and compare with the estimated results of ordinary least squares (OLS), temporally weighted regression (TWR) and geographically weighted regression (GWR) models to verify the applicability of the GTWR model; the relevant model results are shown in Table 4.

GTWR, TWR and GWR are concerned with spatio-temporal non-stationarity, temporal non-stationarity, and spatial non-stationarity, respectively, leading to differences in the fitting results of their models. Based on the combined values of R^2^, adjusted R^2^, and AICc, the GTWR model has the highest R^2^ (0.802), the lowest RSS (8.626), and the lowest AICc value (−909.771). Therefore, the GTWR model with a combination of temporal and spatial non-smoothness is the optimal choice.

## 3. Results

### 3.1. Measurement of Agricultural Water Use Green Efficiency in China

Based on the previous construction of the evaluation index system of green efficiency of agricultural water use, the non-radial, scale-payoff-invariant global Super-SBM model was used in this paper to measure the green efficiency of agricultural water use in China from 1997 to 2020; the results are shown in Table 5.

Generally speaking, the average value of green efficiency of agricultural water use in China from 1997–2020 was only 0.538 and demonstrates a fluctuation trend of first decreasing and then increasing. The national average value increased from 0.538 in 1997 to 0.989 in 2020, and the average annual growth rate was about 3.6%. From 1997 to 2009, the national average value gradually decreased from 0.538 to 0.406, a decline of 24.5%. During this period, China encountered rare natural disasters and financial storms, and the slow development of the agricultural economy directly affected the enthusiasm of farmers for grain planting; the grain output brought by the input of water resources and other factors decreased significantly, and the green efficiency of agricultural water use plummeted. From 2009 to 2020, the national average increased sharply from 0.406 to 0.989, with an increase of 143.6%. During this period, the extensive agricultural production mode, which is highly dependent on the inputs of resource and energy, gradually changed to the green agricultural production mode of “low pollution, low consumption and high output”, and the green efficiency of agricultural water use was greatly improved. From 2015 to 2020, China’s agricultural water use green efficiency remained at a high level, the whole society comprehensively promoted the efficient development and economical utilization of water resources, and agricultural water green efficiency was significantly improved.

In terms of regions, the green efficiency in the eastern region was the highest (0.594), which was above the national average (0.538). It gradually decreased from 0.542 in 1997 to 0.458 in 2009, and then rapidly increased to 1.191 in 2020, with an average annual growth rate of about 5.2%. The green efficiency in the western region was the second highest (0.522), and it gradually decreased from 0.617 in 1997 to 0.396 in 2009, then rapidly increased to 0.882 in 2020, with an average annual growth rate of about 5.2%. The green efficiency in the central region was the lowest (0.491), and it gradually decreased from 0.438 in 1997 to 0.356 in 2009, then rapidly increased to 0.873 in 2020, with an average annual growth rate of about 4.3%.

In terms of cities, there are significant regional differences regarding the green efficiency of agricultural water use in China. Among them, the regions with higher green efficiency are Shanghai, Tibet, Jilin, Tianjin, Heilongjiang, Beijing and Jiangsu, where the average value was greater than 0.7 during the study period, and most of them belong to the developed eastern regions, while the green efficiencies in Hubei, Shanxi, Anhui, Hebei, Xinjiang and Gansu were lower, with the average value less than 0.4. In these regions, Hubei and Hebei are traditional agricultural provinces with more serious agricultural water consumption, while Xinjiang and Gansu have relatively scarce water resources and harsher ecological and climatic conditions, which to a certain extent will increase agricultural water consumption and thus reduce the green efficiency. The agricultural irrigation modes in Shanxi and Anhui are relatively regressive, mostly using large water irrigation and string irrigation, resulting in serious water waste and low green efficiency. In addition, from the perspective of rising range, the green efficiency in all regions except Ningxia in 2020 was greatly improved compared with 2000, and the rising ranges of different regions were different. Shanxi, Beijing and Yunnan rank at the top, and their rising rates were 368.8%, 343.9% and 282.6%, respectively. The rising rates in Shandong, Henan, Fujian and Hebei are also obvious, and the rising rates were all over 200.0%. Only Ningxia’s rising rate in the study period was negative, that is, the green efficiency declined.

### 3.2. Evolution Trend of Green Efficiency of Agricultural Water Use in China

**Figure 2:**
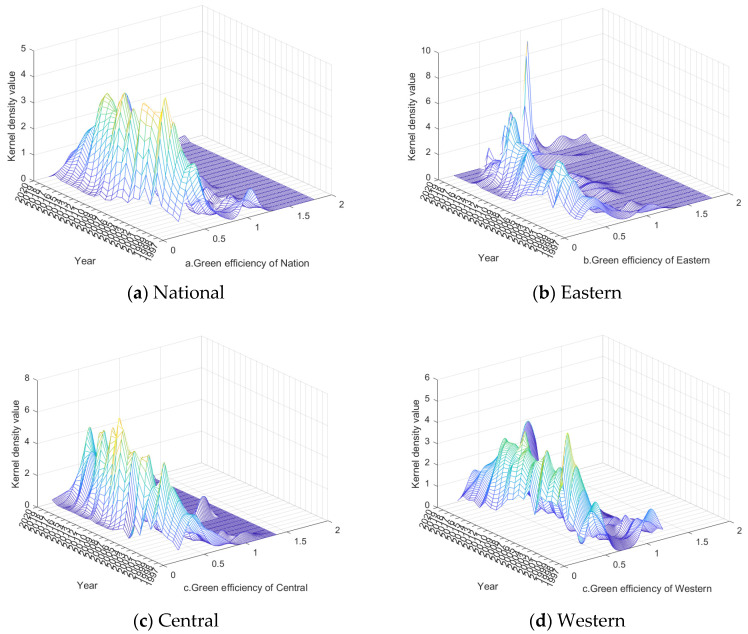
Evolution trends of green efficiency of agricultural water use in China.

The kernel density estimation model was used to reveal the evolution trend of green efficiency of agricultural water use in China (Figure 2). As can be seen from Figure 2a, the position of the main peak shifts first to the left and then to the right, indicating that the green efficiency experienced an evolution trend of “first decreasing and then increasing”, and this feature is basically consistent with the trend of the average curve of the national green efficiency in the previous section. During the study period, the overall curve demonstrates a double peak, and the side peak gradually decreases until it almost disappears in 2020, indicating a weak polarization of green efficiency of agricultural water use in China; this phenomenon gradually disappeared.

As can be seen from Figure 2b–d, the main peak position of green efficiency in the eastern and central regions experiences a complex left–right movement trend. Among them, the eastern region moves more than the western region, where a right-trailing phenomenon is more obvious, individual years present a multimodal form that is flat and wide, and the side peaks increase gradually until reaching their maximum value in 2020, indicating that the eastern region changed rapidly during the sample period. A multi-polar phenomenon appears in some years, and the spatial gap is gradually expanding, most notably until 2020. The peak value of the main peak in the central region is higher than that in the eastern region, showing a double-peak shape, and the value of the side peak is lower, indicating that although there were regional differences of green efficiency in the central region, the polarization phenomenon was relatively weak, and the overall situation was relatively stable. The position of the main peak of the kernel density curve in the western region generally shows a right-shifting trend, gradually transitioning from a double peak to a single peak, and almost disappears until 2020, which indicates that the green efficiency in the western region was continuously improved, and the polarization phenomenon gradually weakened until it disappeared.

### 3.3. Regional Differences of Agricultural Water Use Green Efficiency in China

As can be seen from the above, there was a weak differentiation phenomenon regarding the green efficiency of agricultural water use of different regions. The reasons for such regional differences will be explored in detail as follows.

#### 3.3.1. Overall and Intra-Regional Differences

**Figure 3:**
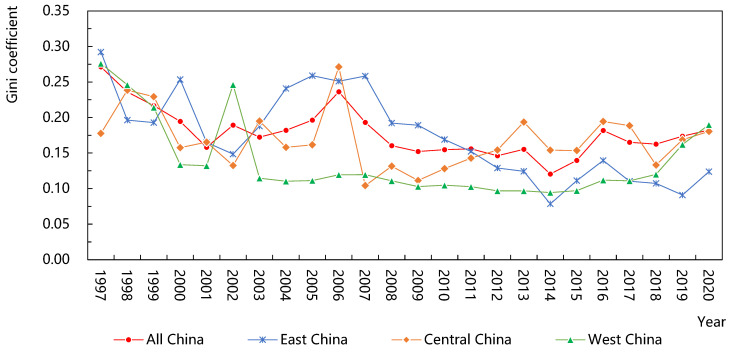
Changes in Dagum Gini coefficient in China and its regions.

As shown in Figure 3, the spatial differences in the green efficiency of agricultural water use in China have fluctuated and declined in general; the internal average difference was 0.18; and the average annual growth rate was about −1.4%, especially in 2006–2008 and 2013–2014, with the decline rates as 32.1% and 22.4%, respectively, which shows that the spatial gap of the green efficiency in different regions was narrowing and had obvious convergence characteristics.

From the perspective of sub-regions, the eastern region was basically consistent with the overall change trend, demonstrating a trend of fluctuation and decline; its internal average gap was the largest (0.173), and its average annual growth rate was also the largest −2.5%), showing significant internal convergence characteristics as a whole. The average gap within the central region was the second (0.168), and the average annual growth rate was positive and the smallest (0.1%), showing an “M-shaped” fluctuation trend of “upward-downward-upward-down”, indicating that there was a weak polarization phenomenon in the central region. The average gap within the western region was the smallest (0.139), with an average annual growth rate of −1.4%, and it maintained a slow and stable differentiation trend from 2003 to 2015. On the whole, it was far less than the national average gap, indicating a high degree of coordination within the western region, which is consistent with the conclusion above.

#### 3.3.2. Regional Differences

**Figure 4:**
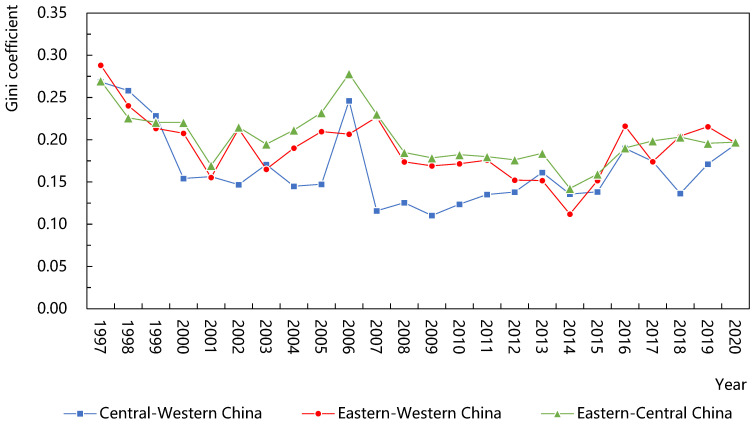
Variation in Dagum Gini coefficient between regions.

As shown in Figure 4, the characteristics of regional differences could be analyzed from two aspects: the size and the trend.

From the size, there were slight differences in the green efficiency of agricultural water use among the eastern, central and western regions. The average gap between the eastern and central regions was the largest (0.20), followed by the eastern and western regions (0.19), and the average gap between the central and western regions was the smallest (0.17). Compared with the central and western regions, the eastern region had the advantage of unique economic and geographical conditions, advanced agricultural irrigation technology and the transformation of resource inputs to intensive type, making the eastern region have higher green efficiency. Because of the extensive input mode of agricultural resources and the low level of agricultural science and technology in the central region, the loss of agricultural water was large and the green efficiency was low, so the gap between the eastern and central regions was the largest.

From the trend, the Dagum Gini coefficients between the eastern and the western, eastern and central, and central and western regions show a strong homogeneity, with a “W-shaped” fluctuation trend of “upward-downward-upward-down”. In addition, the gap among regions is constantly narrowing, and the average annual growth rates are −1.4%, −1.2%, and −1.2%, respectively, indicating that the green efficiency between the eastern and the central regions as well as the eastern and the western regions formed a significant Matthew effect, and the polarization phenomenon was more prominent. Therefore, the eastern region should maintain the first-mover advantage and promote the spatial spillover of agricultural irrigation technology, improve experience-sharing and help mechanisms, and give full play to the exemplary demonstration effect of the eastern region.

#### 3.3.3. Difference Sources and Contribution Decomposition

**Figure 5:**
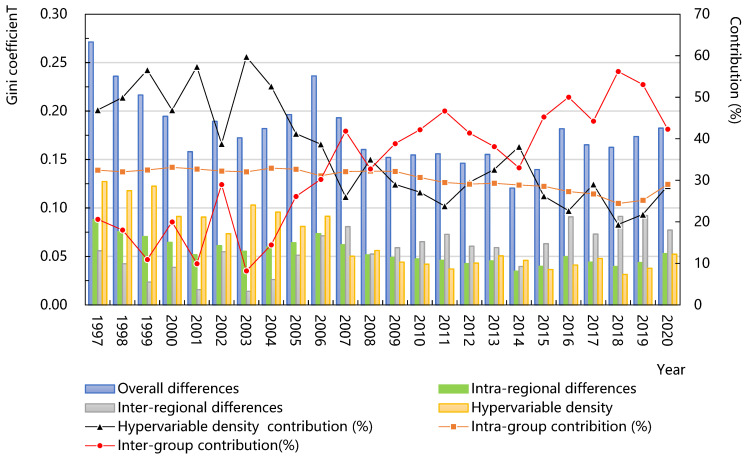
Decomposition of and contribution of the gap between the eastern, central and the western regions.

Figure 5 shows the difference sources and decomposition results of the green efficiency of agricultural water use. It shows that the average contribution rate of super-variable density is the largest (36.6%), larger than the average contribution rate between the regions (33.1%) and within the regions (30.4%), and constitutes the main source of the difference at the national level, indicating that there was a certain degree of crossover between the eastern, central and western regions. Some regions had similar efficiency levels, which reflects the low clustering degree and large difference level. From the perspective of variation trend, the contribution rate within the regions did not change much and remained about 30.4%, while the contribution rate between the groups and super variable density demonstrate two opposite trends of “W-shaped” and “M-shaped”, respectively. Before 2006, the contribution rate of super-variable density was far greater than the others, and after 2006, the difference contribution rate between regions was far greater than the others, indicating that the differences in economic development level, science and technology level, and infrastructure construction affected the green efficiency of agricultural water use to a certain extent. Therefore, while staying focused on solving the gap between regions, we should not neglect the construction of agricultural infrastructure in relatively underdeveloped regions; actively promote the reform of the quality, efficiency and impetus of agricultural development in each region; strive to improve the level of agricultural science and technology; and build a region with strong modern agriculture.

### 3.4. Analysis of Influencing Factors of Agricultural Water Use Green Efficiency in China

#### 3.4.1. Analysis of Influencing factors of the Green Efficiency of Agricultural Water Use in China

The GTWR model was used to obtain the local estimation results from 1997 to 2020. Due to space reasons, only the parameter estimates of the influencing factors in 2020 were selected to analyze their spatial differences. The results are shown in Table 6.

From the perspective of natural factors, the increase in water resource endowment had a bidirectional impact on the improvement of green efficiency, with a greater positive impact on the western region and a greater negative impact on the central and eastern regions. The reason for this is that due to the special geographical environment in the western region, with less precipitation and high evaporation intensity, the loss of agricultural water use was great and the green efficiency was low. However, the balance of water supply and demand in the central and eastern regions and the increase in water resource endowment led to ineffective supply and reduced the green efficiency.

From the perspective of socio-economic factors, the estimated values of the urbanization level parameters were all positive except for in Inner Mongolia, where they were negative, indicating that improvement in the urbanization level had a promoting effect on the improvement of green efficiency. The estimated values of industrialization level parameters were all negative except for in Heilongjiang, where they were positive, indicating that the improvement in industrialization level had a restraining effect on the improvement of green efficiency. The increase in agricultural water use intensity had a bidirectional effect on the improvement of green efficiency, with a greater positive effect on the western region and a greater negative effect on the eastern region. The reason for this is that the economy of the eastern region was relatively developed, the living standard of the residents was higher and the demand for agricultural products such as meat, eggs and milk was much higher. However, the water resource demand of animal husbandry was much higher than that of the planting industry, and the environmental pollutants were greater. Therefore, with the increase in agricultural water intensity, the green efficiency of agricultural water use in the eastern region decreased.

From the perspective of policy factors, the increase in the agricultural financial expenditure ratio had a bidirectional impact on the improvement of green efficiency, with a greater positive impact on the western region and a greater negative impact on the eastern region. The reason for this is that the economic development level of the western region was low, and there was still a large space for investment in the construction of farmland irrigation projects. Increasing the agricultural financial expenditure ratio can improve regressive water-saving irrigation facilities, thereby reducing the unnecessary loss of water resources in the irrigation process and improving the agricultural water use green efficiency. However, the eastern region had a high level of economic development and pursued a high yield of agricultural products in agricultural production, thus increasing the agricultural financial expenditure ratio to purchase a large number of fertilizers and pesticides in order to improve the yield of agricultural products, which led to serious agricultural non-point source pollution and reduced agricultural water use green efficiency. Therefore, as the agricultural financial expenditure ratio increased, the green efficiency in the western region increased, while the green efficiency in the eastern region decreased.

From the perspective of scientific and technological factors, improvement at the water-saving level had a bidirectional impact on the improvement of green efficiency, with a greater positive impact on the eastern region and a greater negative impact on the western region. The reason for this is that the population quality in the eastern region was high, the modern irrigation technology was mastered quickly and the penetration rate was high. Therefore, improvement at the water-saving level would promote the improvement of green efficiency. However, in western China, due to drought and lack of rainfall and relatively regressive irrigation facilities, the effective irrigation area was too large, which might have led to the forced evaporation of part water resources and reduced the green efficiency.

From the perspective of environmental factors, the improvement of food cultivation structure had a bidirectional impact on the improvement of green efficiency, with a greater positive impact on the western region and a greater negative impact on the eastern region. The reason for this is that some of the main agricultural production areas in the eastern region were located in hilly and mountainous areas, where the level of agricultural mechanization was low and the grain production required higher labor costs, resulting in a lower output value of water resources of grain crops per unit.

From the perspective of external opening factors, the improvement of agricultural trade had a bidirectional impact on the improvement of green efficiency, with a greater positive impact on the eastern region and a greater negative impact on the western region. The reason for this is that most of the eastern regions were distributed along the coast and had a relatively high level of economic development, and foreign trade was relatively convenient and developed. Therefore, by taking advantage of their geographical advantages, the eastern regions reduced consumption and water pollution and improved the green efficiency by importing agricultural products.

#### 3.4.2. The Spatial Evolution Characteristics of Each Influencing Factor

**Figure 6:**
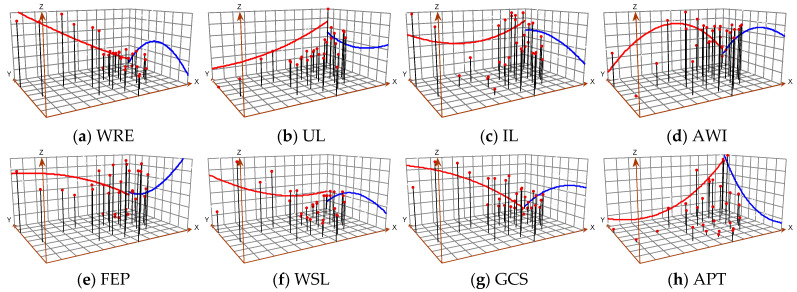
Spatial evolution characteristics of each influencing factor from 1997 to 2020. Note: The X-axis represents the direction of east longitude increments, that is, from west to east; the projection of XZ plane (red line) represents the variation trend of each influencing factor in different regions from west to east. The Y-axis represents the direction of north latitude increments, that is, from south to north; the projection of YZ plane (blue line) represents the variation trend of each influencing factor in different regions from south to north; and the Z-axis represents the green efficiency of agricultural water use in each region.

In order to further study the spatial evolution characteristics of the factors affecting the green efficiency of agricultural water use, the “trend analysis” tool of ArcGIS 10.8 was used to draw a three-dimensional perspective view with the estimated height of the parameters of the factors (Figure 6), project the points (north and west by default) on the plane in two directions of the map plane, and fit a polynomial to obtain a trend line to check the global trend of factors affecting the agricultural water use green efficiency. In Figure 5, the X-axis represents the direction of east longitude increments, that is, from west to east; the projection of the XZ plane (red line) represents the variation trend in each influencing factor in different regions from west to east. The Y-axis represents the direction of north latitude increments, that is, from south to north; the projection of the YZ plane (blue line) represents the variation trend in each influencing factor in different regions from south to north; and the Z-axis represents the green efficiency of agricultural water use in each region.

From the X-direction (meridional direction), the influence of various factors on green efficiency demonstrates different characteristics from west to east. The projection (red line) of urbanization level, water resource endowment, agricultural financial expenditure ratio and food cultivation structure on the XZ plane shows a curve with approximately fixed slope, indicating that the influence of the above factors on the green efficiency of agricultural water use demonstrates a linear trend from west to east. Among them, as the influence of urbanization level on the green efficiency of agricultural water use increases from west to east, the greater the influence on the eastern region. However, the influence of water resource endowment, agricultural financial expenditure ratio and food cultivation structure on the green efficiency of agricultural water use decreases from west to east, and the influence degree of water resource endowment is stronger. The projection (red line) of water-saving level, industrialization level and agricultural trade on the XZ plane is a smooth positive “U” curve, and the slope of the curve decreases first and then increases, indicating that its influence on the green efficiency of agricultural water use decreases first and then increases from west to east and the influence is greater in the eastern region. The projection of agricultural water use intensity on plane Z (red line) is a smooth inverted U-shaped curve, and the slope of the curve decreases first and then increases, indicating that its influence on the green efficiency of agricultural water use increases first and then decreases from west to east and its influence is greater in the western region. To sum up, all factors were sensitive to regional differences in the impact of green efficiency, and different factors had different sensitivities.

From the Y direction (zonal direction), the influence of various influencing factors on the green efficiency still shows different characteristics. The projection of urbanization level, agricultural financial expenditure ratio and agricultural trade on the YZ plane (blue line) is a smooth positive “U” shaped curve, and the slope of the curve decreases first and then increases, indicating that its influence on the green efficiency of agricultural water use decreases first and then increases from north to south and has the least influence on the central region, but the agricultural financial expenditure ratio and agricultural trade do not have obvious “U” shaped characteristics. The projection (blue line) of water resource endowment, industrialization level, agricultural water intensity, water-saving level and food cultivation structure on the YZ plane is a smooth inverted U-shaped curve, and the slope of the curve decreases first and then increases, indicating that its influence on the green efficiency of agricultural water use increases first and then decreases from north to south and the influence is greater in the northern region. In summary, the slope of the projection curve of all influencing factors on the YZ plane (blue line) changes more than that on the XZ plane (red line) and the U-shaped feature is more obvious, indicating that the sensitivity of the influence of all factors on the green efficiency along the north–south axis is stronger than that along the east–west axis. In addition, compared with other influencing factors, the cluster projection lines of agricultural water intensity, industrialization level and urbanization level are more dense in the XZ plane, indicating that they are the most sensitive to the green efficiency of agricultural water use.

#### 3.4.3. The Temporal Evolution Characteristics of Each Influencing Factor

**Figure 7:**
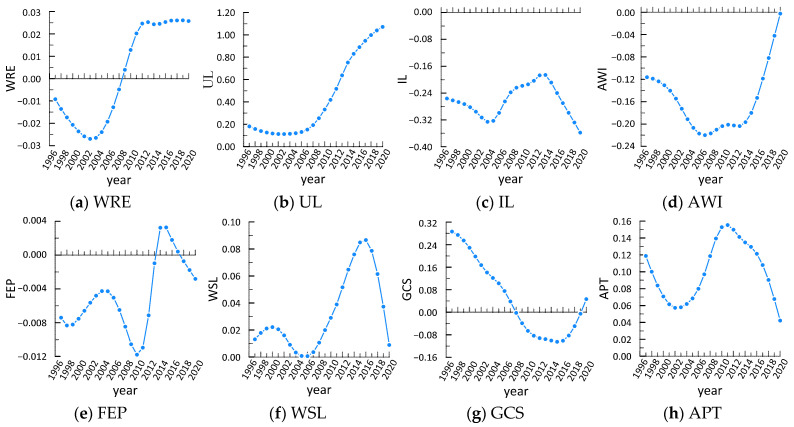
Temporal evolution characteristics of each influencing factor from 1997 to 2020. Note: The X-axis represents year and the Y-axis represents the coefficients of each influencing factor.

In order to further study the temporal evolution characteristics of parameter estimates of the factors affecting the green efficiency, this paper sorts out the average parameter estimates of the impacts of various variables on green efficiency in China from 1997 to 2020, as shown in Figure 7.

From the perspective of natural factors (Figure 6a), the impact of water resources endowment changed from negative to positive, with the trough value as −0.027 in 2004 and the peak value as 0.026 in 2017, with a total span of 0.053 and a positive development trend, indicating that the increase in water resources per capita had a promoting effect on improvement of the green efficiency.

From the perspective of socio-economic factors (Figure 6b–d), urbanization level always had a positive effect on green efficiency, while industrialization level and agricultural water use intensity always had a negative effect on green efficiency. This is because the development of urbanization was accompanied by the inflow of advanced technology, capital and talents into the vast rural areas. On the one hand, this provided more scientific and technological factors for the transformation of traditional agriculture and promoted the transformation of traditional extensive agricultural production into a modern intensive and efficient production mode. On the other hand, this improved the quality of the rural population and enhanced their water-saving awareness and environmental protectiveness, so that the same or even more agricultural products could be produced and the green efficiency could be improved on the premise of reducing resource input and pollution emission. However, there were still many areas that still used traditional extensive irrigation modes such as flood irrigation in agricultural production, resulting in serious agricultural water waste and reducing the green efficiency. In addition, the improvement in industrialization level accelerated the transfer of rural resources, and the resource constraints of agricultural production became increasingly tight. In terms of water resources, non-agricultural water use increased rapidly, and the phenomenon of crowding out agricultural water occurred frequently, which might have reduced agricultural output and reduced the green efficiency.

From the perspective of policy factors (Figure 6e), the influence of the agricultural financial expenditure ratio was negative before 2013 and positive from 2013 to 2017, then turned negative after 2017, with the trough value as −0.012 in 2010 and the peak value as 0.003 in 2014, a total span of 0.015, and a negative development trend, indicating that increasing the agricultural financial expenditure ratio increased farmers’ overuse of agricultural means of production such as diesel oil, chemical fertilizers and pesticides, which brought agricultural non-point source pollution and reduced the green efficiency of agricultural water use.

From the perspective of scientific and technological factors (Figure 6f), the water-saving level always had a positive effect on green efficiency, indicating that strengthening the construction of irrigation facilities moderately and improving the effective irrigation area were the main ways to ensure grain yield and improve the green efficiency of agricultural water use.

From the perspective of environmental factors (Figure 6g), the impact of food cultivation structure was positive before 2007 and negative from 2007 to 2019, then turned positive after 2019. Generally speaking, the output value of water resources per unit of cash crops was greater than food crops. Therefore, the higher the planting area of food crops, the lower the green efficiency. However, with gradual improvements in the national food security strategy, we are now required to not unilaterally pursue economic benefits, adjust the food cultivation structure rationally and advocate for intensive irrigation, not only to take into account green efficiency, but also ensure national food security.

From the perspective of external opening factors (Figure 6h), the impact of agricultural trade was always positive, but the effect has gradually weakened in recent years. When the contradiction between grain production and water resources became increasingly prominent, agricultural trade became an effective way to solve the agricultural water problem. Grain produced in areas with high water efficiency was transported to agricultural production areas with low water efficiency through agricultural trade, and the overall water efficiency was improved. However, under the impact of COVID-19, the balance pattern of global food production and trade was broken. Some grain exporting countries have adopted export bans or restrictions, which hampered agricultural trade and weakened the effect of improving the overall green efficiency.

## 4. Conclusions

Improving the green efficiency of agricultural water use is a key way to promote the sustainable utilization of agricultural water resources and the sustainable development of economy and society. The current paper expands the theory and index system of the agricultural water footprint and regional agricultural water use green efficiency assessment, on top of exploring the spatial–temporal heterogeneity of impact on agricultural water use green efficiency from the aspects of nature, economy, policy, technology, environment and opening to the outside world. We found that the green efficiency of agricultural water use in China demonstrated a fluctuation trend of first declining and then rising from 1997 to 2020; the average efficiency dropped from 0.538 in 1997 to 0.406 in 2009, then rose rapidly to 0.989 in 2020, with an average annual growth rate of about 3.6%. From a regional perspective, the green efficiency of agricultural water use in the eastern region was the highest (0.594), above the national average (0.538), followed by the western region (0.522), and it was lowest in the western region (0.491), with significant regional differences. In addition, the regional differences of agricultural water use green efficiency in China demonstrated a fluctuating downward trend. The Gini coefficient fluctuated from 0.271 in 1997 to 0.182 in 2020, with an average annual growth rate of about −1.4%. The main source of this regional difference was super-variable density, whose average contribution rate was about 36.6%, which was larger than the average contribution rate between regions (33.1%) and within regions (30.4%). In terms of inter-regional differences, the average difference between the eastern region and the central region was the largest, with a Gini coefficient of 0.20. In terms of intra-regional differences, the average difference within the eastern region was the largest, with a Gini coefficient of 0.173. We also found that the influence of urbanization level, water-saving level and agricultural trade on the green efficiency of agricultural water use was always positive and the influence of industrialization level was always negative; among them, the urbanization level, water-saving level, and industrialization level had a greater impact on Northeast China, and agricultural trade had a greater impact on Southeast China.

## 5. Discussion

Improving the green efficiency of agricultural water use is a key way to promote the sustainable utilization of agricultural water resources and the high-quality development of the economy and society. Since the State Council promulgated and implemented “the opinion on implementing the strictest water resources management system” in 2011, the government has attached great importance to “water resources management” and comprehensively promoted agricultural modernization and water-saving society construction, and the green efficiency of agricultural water use in China has been improved significantly. However, there was a weak differentiation phenomenon regarding the green efficiency of different regions, where the eastern region was the highest, because China’s “South-to-North Water Diversion Project” has played an important role in the eastern region and helped improve the agricultural water conditions, thus improving the green efficiency of agricultural water use. In addition, the eastern region has a more developed level of economic development than the central and western regions, with more investment in water-saving irrigation technology and facilities, and higher green efficiency. However, the internal average gap within the eastern region was the largest. On the one hand, some first-tier regions such as Beijing, Tianjin, Shanghai took the lead in actively responding to China’s national water-saving action; the local water-saving work was carried out comprehensively and with large investment, thus, the green efficiency improved rapidly. On the other hand, for Shandong, Hebei and other major agricultural provinces, while increasing crop yield, a large number of agricultural chemicals (fertilizers, pesticides) were applied, resulting in serious water pollution and low green efficiency. The central and western regions were regressive in development and suffered a large loss in agricultural water use; there was great room for improving the green efficiency of agricultural water use.

China is a vast territory. Different regions had different resource endowments, economic levels, agricultural technology levels and environmental policies, resulting in different green efficiencies of agricultural water use. The influence of urbanization level, water-saving level and agricultural trade on the green efficiency of agricultural water use was always positive and the influence of industrialization level was always negative; among them, the urbanization level, water-saving level, and industrialization level had a greater impact on Northeast China, and agricultural trade had a greater impact on Southeast China. Therefore, in order to comprehensively improve the green efficiency of agricultural water use in China, the following policy recommendations are put forward: (1) Deepen the reform of the household registration system, promote the two-way free flow of urban and rural factors and comprehensively improve the quality of urbanization. However, due to the imperfect household registration system, migrant workers cannot enjoy the same treatment as urban residents in medical care, education and employment, which seriously hinders the process of urbanization. Therefore, while deepening the reform of the household registration system, all regions should improve the construction of rural public service facilities, so that urban and rural residents can enjoy equal rights. Especially for the northeast region, accelerate the reform of the household registration system, encourage the rural population to enter the urban regions to expand the frontier of science and technology, improve the ability to transform scientific and technological achievements and promote the two-way free flow of urban and rural factors, which will comprehensively improve the quality of urbanization. (2) Strengthen the construction of water conservancy and irrigation facilities and increase the effective irrigation area. The implementation of high-efficiency water-saving irrigation is an important strategic measure to alleviate the crisis of water shortage, improve the efficiency of water resource utilization and promote sustainable economic and social development. However, water conservancy and irrigation facilities have the attribute of “public goods” and are limited by the size of land and use. For small-scale farmers, the cost is high and the investment is difficult to recover in the short term, which limits the effectiveness of water saving. Therefore, all regions should increase financial support for the construction of water-saving irrigation facilities and promote water-saving irrigation technology on a large scale. In particular, for the northeast region, where water resources are relatively short, the government should increase financial support for the development of water-saving irrigation, strengthen the construction of irrigation facilities for water conservancy and irrigation facilities and promote agricultural water-saving by improving the effective irrigation area. (3) Accelerate the promotion of trade in agricultural products and ease the pressure on resources and the environment. Through the import and export of agricultural products, the import and export of water resources are also carried out immediately. On the one hand, this makes up for the large amount of water resources consumed by food production in water-scarce areas; on the other hand, it indirectly transfers or alleviates local water pollution and improves the green efficiency. Therefore, each region should arrange agricultural production according to its water resource status. For the arid regions in the west and North China, they should import more agricultural products with high water consumption such as soybeans and wheat and increase the import of water resources to alleviate water shortage stress. In addition, the southeast coastal areas should rely on their natural and economic geographical advantages to export more energy, instrumentation, machinery manufacturing and other products with relatively small water consumption and high added value. (4) Coordinate the development of industry and agriculture to alleviate the contradiction between supply and demand of agricultural water resources. With the continuous advancement of industrialization, part of agricultural water is gradually crowded out by industrial water and urban water. The contradiction between supply and demand of agricultural water resources is intensified and agricultural output is reduced, which has a negative impact on food security to a large extent. Especially for Northeast China, under the background of accelerating the comprehensive revitalization of the old industrial base in Northeast China, the level of industrialization has been significantly improved, resulting in serious water pollution and posing severe challenges to the green development of agricultural water. Therefore, in order to alleviate the crowding out and pollution of agricultural water in the process of industrialization, local governments should coordinate the development of industry and agriculture and alleviate the contradiction between supply and demand of agricultural water resources by implementing such laws and policies that will control of the use of agricultural water resources, compensate for agricultural water transfer and prevent agricultural water pollution.

In this manuscript, the green efficiency of agricultural water use remains a preliminary study. There are a few problems that have yet to be researched: (1) When constructing the evaluation index system, the expected output only selected representative indicators such as the total output value of the primary industry and the total grain output. However, the development of an agricultural economy should be comprehensive, including science, education, personnel, medical treatment and other aspects. Due to the lack of data, these aspects were not included in the evaluation index system, and these data should be gradually replenished in future research. (2) Due to space limitations, this manuscript only used the Super-SBM model to measure the green efficiency of agricultural water use. The Malmquist–Luenberger index was not used to reveal the reasons for the efficiency change, that is, whether it was caused by pure technological progress or scale technological progress. This is crucial for understanding the regional differences regarding the green efficiency of agricultural water use, and this part should be supplemented in future research.

## Figures and Tables

**Table 1:** The index system of green efficiency of agricultural water use.

Indicator Category	Selection Basis	Indicators	Unit	Literatures Emerged/Studied
Input Variables	Land	Crop seeding area	10^3^ hm^2^	[20,39]
	Labor	Primary industry labor force	10^4^	[2,28]
	Agricultural capital	Total power of agricultural machinery	10^4^ kw	[28,39]
	Agricultural water resources	Agriculture blue and green water footprint	m^3^	[17,40,41]
	Technology	Fertilizer application discounted amount	kg	[2,20]
		Pesticide application amount	kg	[2,20]
Output Variables	Expected output	Total output value of primary industry (discounted to 1997 constant prices)	10^8^ RMB	[20,42]
		Total grain production	kg	[28,38]
	Unexpected output	Agricultural grey water footprint	m^3^	[38,43,44,45]
		Agricultural carbon emissions	kg	[46,47]

**Table 2:** Influencing factors of the green efficiency of agricultural water use.

Factor Classification	Explanatory Variables and Units	Reference Sources
Natural factors	(WRE) Water resource endowment: per capita water resources (m^3^/person)	[48]
Socio-economic factors	(UL) Urbanization level: urban population/total population (%)	[49]
	(IL) Industrialization level: industrial value added/regional GDP (%)	[50]
	(AWI) Agricultural water use intensity: total agricultural water use/total water use (%)	[48]
Policy factors	(FEP) Agricultural financial expenditure ratio: expenditure on agriculture, forestry and water affairs/local general public budget expenditure (%)	[2]
Scientific and technological factors	(WSL) Level of water-saving agriculture: arable (effective) irrigated area/crop sown area (%)	[25]
Environmental factors	(GCS) Food cultivation structure: area sown with food crops/area sown with crops (%)	[38]
External opening factors	(APT) Agricultural trade: total import and export of agricultural products/regional GDP (%)	[33]

**Table 3:** Results of multicollinearity test.

Model Variables	WRE	UL	IL	AWI	FEP	WSL	GCS	APT	Average VIF
VIF	1.61	2.47	1.14	2.08	2.38	1.33	1.73	2.89	1.95
1/VIF	0.62	0.41	0.88	0.48	0.42	0.75	0.58	0.35	0.56

**Table 4:** Estimation results and comparison of parameters of each model.

Variables	OLS Model	TWR Model	GWR Model	GTWR Model
R^2^	0.401	0.580	0.660	0.802
R^2^ adjusted	—	0.576	0.656	0.800
RSS	26.088	18.298	14.833	8.626
AICc	−363.429	−545.588	−650.257	−909.771
Sigma	—	0.157	0.141	0.108
Bandwidth	—	0.115	0.115	0.115

**Table 5:** Results of green efficiency of agricultural water use in China from 1997 to 2020.

Grouping	Region	Year									Average
		1997	2000	2003	2006	2009	2012	2015	2018	2020	
Eastern region	Beijing	0.404	0.374	0.344	1.011	0.523	0.538	0.583	0.817	1.792	0.709
	Tianjin	1.016	1.001	0.350	0.423	0.440	0.495	0.505	1.005	1.460	0.744
	Hebei	0.233	0.208	0.201	0.297	0.308	0.412	0.427	0.508	0.752	0.372
	Liaoning	0.352	0.297	0.348	0.527	0.389	0.621	0.696	0.778	1.001	0.557
	Shanghai	1.012	0.614	0.669	0.790	1.015	0.882	0.750	1.022	1.533	0.921
	Jiangsu	1.031	0.350	0.299	0.450	0.549	0.698	0.856	1.008	1.137	0.709
	Zhejiang	0.329	0.285	0.244	0.249	0.329	0.443	0.506	0.592	1.057	0.448
	Fujian	0.323	0.298	0.278	0.268	0.360	0.500	0.586	0.764	1.070	0.494
	Shandong	0.273	0.237	0.211	0.311	0.319	0.390	0.500	0.823	1.034	0.455
	Guangdong	0.406	0.354	0.324	0.319	0.378	0.509	0.520	0.730	1.082	0.514
	Hainan	0.578	0.482	0.464	0.406	0.421	0.547	0.613	0.787	1.181	0.609
Average		0.542	0.409	0.339	0.459	0.458	0.549	0.595	0.803	1.191	0.594
Central region	Shanxi	0.245	0.204	0.231	0.328	0.272	0.320	0.323	0.424	1.147	0.388
	Inner Mongolia	0.427	0.337	0.327	0.667	0.314	0.407	0.427	0.499	0.501	0.434
	Jilin	0.716	0.564	0.664	1.046	0.484	0.771	0.832	0.657	1.005	0.749
	Heilongjiang	0.556	0.374	0.370	1.121	0.479	0.620	0.686	1.007	1.375	0.732
	Anhui	0.355	0.278	0.236	0.372	0.310	0.371	0.406	0.510	0.641	0.387
	Jiangxi	0.577	0.430	0.399	0.416	0.380	0.399	0.511	0.576	0.901	0.510
	Henan	0.304	0.258	0.212	0.341	0.287	0.335	0.414	0.537	1.057	0.416
	Hubei	0.385	0.300	0.272	0.348	0.321	0.385	0.440	0.504	0.587	0.394
	Hunan	0.379	0.335	0.299	0.325	0.355	0.434	0.478	0.463	0.638	0.412
Average		0.438	0.342	0.334	0.552	0.356	0.449	0.502	0.575	0.873	0.491
Western region	Guangxi	0.356	0.291	0.276	0.266	0.303	0.462	0.493	0.551	0.640	0.404
	Chongqing	1.029	0.436	0.411	0.413	0.449	0.505	0.540	0.642	1.144	0.619
	Sichuan	1.003	0.413	0.412	0.422	0.392	0.480	0.503	0.630	1.054	0.590
	Guizhou	0.478	0.433	0.379	0.316	0.336	0.332	0.457	0.700	1.058	0.499
	Yunnan	0.311	0.315	0.292	0.343	0.300	0.333	0.371	0.456	1.191	0.435
	Shaanxi	0.336	0.307	0.279	0.346	0.339	0.412	0.447	0.456	1.036	0.440
	Gansu	0.342	0.287	0.287	0.364	0.284	0.326	0.296	0.398	0.440	0.336
	Qinghai	0.417	0.363	0.407	0.460	0.478	0.536	0.563	0.708	1.152	0.565
	Ningxia	1.122	0.630	0.512	0.597	0.452	0.457	0.468	0.545	0.466	0.583
	Xinjiang	0.357	0.315	0.308	0.440	0.323	0.389	0.374	0.377	0.445	0.370
	Tibet	1.039	1.036	1.007	1.006	0.699	0.704	0.713	0.819	1.078	0.900
Average		0.617	0.439	0.416	0.452	0.396	0.449	0.475	0.571	0.882	0.522
National average		0.538	0.400	0.365	0.484	0.406	0.484	0.525	0.655	0.989	0.538

**Table 6:** Parameter estimates of influencing factors of agricultural water use green efficiency in 2020.

Region	Provinces	WRE	UL	IL	AWI	FEP	WSL	GCS	APT	Average
Eastern region	Beijing	0.011	0.309	−0.464	−0.381	−0.054	−0.029	−0.126	0.330	−0.051
	Tianjin	0.009	0.437	−0.514	−0.295	−0.052	−0.002	−0.263	0.334	−0.043
	Hebei	0.006	0.405	−0.452	−0.331	−0.050	−0.031	−0.146	0.306	−0.037
	Liaoning	0.035	0.449	−0.512	−0.361	−0.054	0.148	−0.481	0.445	−0.041
	Shanghai	−0.013	1.412	−0.615	0.179	−0.015	0.155	−0.678	0.297	0.090
	Jiangsu	−0.017	1.256	−0.560	0.112	−0.017	0.136	−0.600	0.282	0.074
	Zhejiang	−0.026	1.591	−0.452	0.213	0.001	0.096	−0.375	0.187	0.154
	Fujian	−0.052	1.806	−0.258	0.270	0.031	−0.004	0.012	0.024	0.229
	Shandong	−0.006	0.874	−0.551	−0.069	−0.035	0.065	−0.492	0.307	0.012
	Guangdong	−0.076	1.541	−0.202	0.295	0.058	−0.120	0.477	−0.198	0.222
	Hainan	−0.072	1.255	−0.297	0.268	0.058	−0.083	0.723	−0.276	0.197
Central region	Shanxi	−0.007	0.677	−0.311	−0.161	−0.030	−0.062	−0.031	0.156	0.029
	Inner Mongolia	0.021	−0.125	−0.344	−0.681	−0.061	−0.055	0.163	0.381	−0.088
	Jilin	0.066	1.086	−0.173	−0.233	−0.016	0.203	−0.339	0.291	0.111
	Heilongjiang	0.155	1.951	0.123	−0.027	0.019	0.110	−0.486	0.072	0.240
	Anhui	−0.033	1.263	−0.413	0.133	−0.007	0.075	−0.339	0.172	0.106
	Jiangxi	−0.065	1.562	−0.231	0.255	0.032	−0.059	0.102	−0.040	0.195
	Henan	−0.026	0.905	−0.413	0.053	−0.020	−0.017	−0.140	0.117	0.057
	Hubei	−0.041	0.975	−0.401	0.150	0.001	−0.071	0.012	−0.006	0.077
	Hunan	−0.060	1.318	−0.249	0.212	0.039	−0.147	0.311	−0.146	0.160
Western region	Guangxi	−0.050	1.401	−0.267	0.228	0.057	−0.182	0.660	−0.265	0.198
	Chongqing	0.017	0.972	−0.477	0.082	0.020	−0.118	0.308	−0.110	0.087
	Sichuan	0.127	1.104	−0.479	0.031	0.014	−0.044	0.242	−0.082	0.114
	Guizhou	0.010	1.360	−0.315	0.150	0.049	−0.177	0.588	−0.233	0.179
	Yunnan	0.128	1.460	−0.525	0.005	0.053	−0.211	0.908	−0.390	0.179
	Shaanxi	0.023	0.670	−0.376	0.029	−0.019	−0.038	0.118	0.015	0.053
	Gansu	0.135	1.141	−0.203	0.248	−0.035	0.164	0.428	−0.028	0.231
	Qinghai	0.200	1.105	−0.662	0.090	−0.024	0.015	0.513	−0.033	0.151
	Ningxia	0.069	0.737	−0.159	0.034	−0.020	0.077	0.029	−0.009	0.095
	Xinjiang	0.168	1.112	−0.023	0.294	0.006	0.182	−0.213	−0.507	0.127
	Tibet	0.160	1.183	−0.324	−0.871	−0.016	0.301	0.560	−0.086	0.113
National	Average	0.026	1.071	−0.358	−0.003	−0.003	0.009	0.047	0.042	0.104

## Data Availability

Data are available on request due to restrictions, e.g., privacy or ethics. The data presented in this study are available on request from the corresponding author. The data are not publicly available due to the strict management of various data and technical resources within the research teams.

## Appendix A

### Water Footprint Model

The agricultural water footprint represents the total amount of fresh water consumed by a country, region or individual when consuming all agricultural products and services within a certain period of time. It is the sum of blue water, green water and grey water footprints. Among them, the blue water footprint refers to the total amount of groundwater and surface water consumed during crop growth, the green water footprint refers to the effective precipitation absorbed and utilized during crop growth and the grey water footprint refers to the amount of fresh water resources used to dilute pollutants generated by fertilizer and pesticide application. The specific calculation formula is as follows:

(A1) $$AWF=AWF_{blue}+AWF_{green}+AWF_{grey}$$

In Equation (A1),  AWF  represents agricultural water footprint (m^3^) and $AWF_{blue}$, $AWF_{green}$ and $AWF_{grey}$ represent agricultural blue water footprint, green water footprint and grey water footprint (m^3^), respectively.

(1) Agricultural blue and green water footprint

In this paper, the agricultural blue and green water footprint include planting, forestry, animal husbandry and fishery, which are calculated according to the “top-down method”. The output of all kinds of products in each province (city, district) and the virtual water content of blue and green water per unit product are multiplied and summed [31]. The calculation formula is as follows:

(A2) $$AWF_{blue}=\overset{n}{\underset{i=1}{\sum }}P_{i}\;\cdot \;VWC_{i}$$

(A3) $$AWF_{green}=\overset{n}{\underset{i=1}{\sum }}P_{i}\;\cdot \;VWC_{i}$$

In Equations (A2) and (A3), $P_{i}$ is the output of the *i* product (kg) and $VWC_{i}$ is the unit blue and green water virtual water content of the *i* product (m^3^/kg); its value adopts the research results of Chapagain and Hoekstra on China in 2004 and the research results of Cheng et al. (Table A1) [35,36,37].

**Table A1:** Virtual water content of blue and green water per unit mass of planting, forestry, animal husbandry and fishery products (m^3^/kg).

Product Category	Product Name	Blue Water	Green Water	Product Category	Product Name	Blue Water	Green Water
Planting	Wheat	0.342	1.277	Planting	Apple	0.133	0.561
	Corn	0.081	0.947	Forestry	Walnut	0.37	4.37
	Rice	0.341	1.146		Chestnut	0.12	1.46
	Millet	0.057	4.306	Animal husbandry	Pork	0.46	4.91
	Sorghum	0.103	2.857		Beef	0.55	14.41
	Soybean	0.07	2.037		Mutton	0.46	8.25
	Potato	0.033	0.191		Poultry	0.31	3.55
	Cotton	0.432	0.755		Rabbit	0.21	3.78
	Oil	0.337	2.489		Egg	0.24	2.59
	Vegetable	0.062	0.22		Milk	0.09	0.86
	Melon	0.002	0.15	Fishery	Aquatic product	0.56	3.57

(2) Agricultural grey water footprint

In this paper, the agricultural grey water footprint ($AWF_{grey}$) is composed of three parts: the grey water footprint of the planting industry ($WF_{grey,\;plant}$), the grey water footprint of the livestock and poultry breeding industry ($WF_{grey,\;live}$) and the grey water footprint of the aquaculture industry ($WF_{grey,\;fishery}$) [38,39,40], and the specific calculation formula is as follows:

(A4) $$AWF_{grey}=WF_{grey,\;plant}+WF_{grey,\;live}+WF_{grey,\;fishery}$$

(3) Grey water footprint of planting industry

In the process of crop growth, a large amount of fertilizers and pesticides are applied. Except for a few residues in the soil that are absorbed and utilized by crops, most of them will enter the water environment with a fixed proportion (leaching rate) along with precipitation or irrigation water, causing pollution of surface runoff and underground water. In view of the large application rate of organophosphate pesticides such as dichlorvos in China and the serious pollution of the ecological environment caused by nitrogen and phosphorus generated by the application of chemical fertilizers [41], in this paper, only the grey water footprint of phosphorus produced by the application of organophosphorus pesticides and the grey water footprint of nitrogen and phosphorus produced by nitrogen fertilizer, phosphorus fertilizer and compound fertilizer are considered. Among them, the application amount of organophosphorus pesticides is replaced by the application amount of pesticide. The ratio of *N*, *P* and *K* in the compound fertilizer is determined according to 1:0.8:0.7 [38,42,43]. The specific calculation formula is as follows:

(A5) $$WF_{grey,\;plant}=max(WF_{grey,\;plant}(TN),\;WF_{grey,\;plant}(TP))$$

(A6) $$WF_{grey,\;plant}=\frac{\alpha \;Appl}{C_{max}-C_{nat}}$$

In Equations (A5) and (A6), $WF_{grey,\;plant}$ is the grey water footprint of the planting industry (m^3^), $WF_{grey,\;plant}(TN)$ is the nitrogen grey water footprint (m^3^) produced by nitrogen fertilizer and compound fertilizer application, $WF_{grey,\;plant}(TP)$ is the phosphorus grey water footprint (m^3^) produced by the application of organophosphorus pesticides, phosphate fertilizers and compound fertilizers; α is the leaching rate, 10% is selected as the leaching rate of nitrogen, 3% as the leaching rate of phosphate fertilizers, and 0.4852% as the leaching rate of organophosphorus pesticides [44]; and Appl represents the pure volume (kg) of organophosphorus pesticides, nitrogen fertilizers, phosphate fertilizers and compound fertilizers. The standard concentration of water quality $C_{max}$ (kg/m^3^) is in accordance with the Class III water quality standard in “Environmental Quality Standard for Surface Water” (GB 3838-2002), and the natural background concentration of various pollutants in the receiving water body $C_{nat}$ (kg/m^3^) refer to the research results of Cheng et al. [36].

(4) Grey water footprint of livestock and poultry breeding industry

Manure, urine and waste water produced by livestock and poultry breeding will seriously deteriorate the quality of surface water and groundwater if not treated effectively. In this paper, feces and urine produced by representative livestock (pigs, cattle, sheep, poultry and rabbits) in the process of breeding are selected as the pollution sources of breeding industry [45,46]. In view of the double counting caused by considering both the quantity of livestock produced and the quantity of livestock stored at the same time, the annual quantity of livestock and poultry is classified according to the breeding cycle of livestock and poultry [47,48]. For cattle and sheep with a feeding cycle of more than or equal to 365 days and pigs, poultry and rabbits with a feeding cycle of less than 365 days, the year-end stock quantity is taken; this method is more objective in measuring the annual livestock and poultry breeding scale [49]. Since TN and COD are relatively high in the manure and urine pollutants of livestock and poultry, and water bodies can dilute TN and COD at the same time, the largest grey water footprint produced by TN and COD pollutants is selected as the grey water footprint of livestock and poultry industry. The specific calculation formula is as follows:

(A7) $$WF_{grey,\;live}=max(WF_{grey,\;live}(TN),\;WF_{grey,\;live}(COD))$$

(A8) $$WF_{grey,\;live}=\frac{L}{C_{max}-C_{nat}}$$

(A9) $$L=\overset{4}{\underset{h=1}{\sum }}N_{h}\times D_{h}(f_{h}\times P_{hf}\times \beta_{hf}+u_{h}\times P_{hu}\times \beta_{hu})$$

In Equations (A7)–(A9): $WF_{grey,\;live}$ represents the grey water footprint of livestock and poultry breeding industry (m^3^); *L* represents the total TN or COD content of livestock and poultry manure entering the water body (kg); *h* represents pigs, cattle, sheep, poultry and rabbits; $N_{h}$ is the feeding quantity of *h* (head or only); $D_{h}$ is the feeding period of *h* (d); $f_{h}$ is the daily fecal volume of *h* (kg/d); $u_{h}$ is the daily urine output of *h* (kg/d); $P_{hf}$ is the pollutant content of *h* unit of feces (kg/t); $P_{hu}$ is the pollutant content of *h* unit of urine (kg/t); $\beta_{hf}$ is the loss rate of pollutants of *h* unit of feces (%); $\beta_{hu}$ is the unit of *h* Contaminant loss rate (%) in urine.

(5) Grey water footprint of aquaculture

With the development of fishery, aquaculture has become an important part of ensuring China’s food security and implementing the strategy of “resource substitution in short supply”. However, as the amount of aquaculture exceeds the allowable ecological capacity considering the massive discharge of residual bait, debris, fish excrement and other substances, the dissolved oxygen content in the water decreases and the ammonia nitrogen content increases, resulting in the deterioration of water quality and environmental pollution of aquaculture water. At the same time, the deterioration of water quality also breeds a large number of viruses, bacteria, etc., which seriously affects the quality and safety of aquaculture products, restricts the sustainable development of aquaculture and is one of the sources of agricultural non-point source pollution [50].

According to relevant statistics, grass carp is the main fish species in freshwater aquaculture in China, *Penaeus vannamei* is the main shrimp species, and mussels are the main shellfish species. Therefore, this paper divides the provinces (cities and districts) of the country into four regions: northeast region, northern region, central region and southern region. Grass carp, *Penaeus vannamei* and river mussels are taken as the research objects, and total nitrogen, total phosphorus and COD are selected as pollution factors to calculate the provincial (city, area) aquaculture grey water footprint. The four large area concrete division and computation formula is as follows (Table A2):

**Table A2:** The specific division of the four major regions.

Region	Province (City, Autonomous Region)
Northeast region	Heilongjiang, Jilin, Liaoning
Northern region	Inner Mongolia, Xinjiang, Qinghai, Gansu, Shaanxi
	Shanxi, Beijing, Shandong, Hebei, Ningxia, Tianjin
Central region	Hubei, Hunan, Sichuan, Henan, Anhui
	Shanghai, Jiangsu, Zhejiang, Jiangxi, Chongqing
Southern region	Guangdong, Hainan, Fujian, Guizhou, Yunnan, Xizang, Guangxi

(A10)
$$WF_{grey,\;fishery}=max(WF_{grey,\;fishery}(TN),\;WF_{grey,\;fishery}(TP),\;WF_{grey,\;fishery}(COD))$$

(A11)
$$WF_{grey,\;fishery}(i)=\frac{L_{fis_{\left(i\right)}}}{C_{max}-C_{nat}}$$

(A12)
$$L_{fis_{\left(i\right)}}=\overset{3}{\underset{h=1}{\sum }}B_{h}\times E_{h}$$

In Equations (A10)–(A12), $WF_{grey,\;fishery}$ is the grey water footprint of the aquaculture industry (m^3^), $WF_{grey,\;fishery}(i)$ is the grey water footprint of the aquaculture industry representing the *i* pollutant (*i*) = TN/TP/COD, $L_{fis_{\left(i\right)}}$ is the pollution load of the *i* pollutant aquaculture industry (kg), $B_{h}$ is the fish farming output (kg), and $E_{h}$ is the pollutant discharge coefficient (g/kg), as shown in Table A3.

**Table A3:** TN, TP and COD discharge coefficients of grass carp, *Penaeus vannamei* and mussel in different regions of China (g/kg).

Pollution Type	Region	*TN*	*TP*	*COD*
Grass carp (g/kg)	Northeast region	0.766	0.209	12.76
	Northern region			
	Central region	7.975	1.569	90.877
	Southern region	5.098	1.188	30.345
Penaeus vannamei (g/kg)	National	1.311	0.106	34.655
Mussels (g/kg)	National	12.264	1.055	60.938

## References

[1] Chung M.G., Frank K.A., Pokhrel Y. (2021). Natural infrastructure in sustaining global urban freshwater ecosystem services. Nature.

[2] Wei J.X., Lei Y.L., Yao H.J., Ge J.P., Wu S.M., Liu L.N. (2021). Estimation and influencing factors of agricultural water efficiency in the Yellow River basin, China. J. Clean. Prod..

[3] United States National Intelligence Council (2012). Global Trends 2030: Alternative Worlds.

[4] National Bureau of Statistics (2021). China Statistical Yearbook 2020.

[5] Ministry of Water Resources, PRC (2021). China Water Resources Bulletin 2020.

[6] Geng Q.L., Ren Q.F., Nolan R.H., Wu P.T., Yu Q. (2019). Assessing China’s agricultural water use efficiency in a green-blue water perspective: A study based on data envelopment analysis. Ecol. Indic..

[7] Schaldach R., Koch J., der Beek T.A., Kynast E., Flörke M. (2012). Current and future irrigation water requirements in pan-Europe: An integrated analysis of socio-economic and climate scenarios. Glob. Planet. Chang..

[8] Multsch S., Elshamy M.E., Batarseh S., Seid A.H., Frede H.G., Breuer L. (2017). Improving irrigation efficiency will be insufficient to meet future water demand in the Nile Basin. J. Hydrol. Reg. Stud..

[9] Caldera U., Breyer C. (2019). Assessing the potential for renewable energy powered desalination for the global irrigation sector. Sci. Total Environ..

[10] Berbel J., Gutiérrez-Martín C., Expósito A. (2018). Impacts of irrigation efficiency improvement on water use, water consumption and response to water price at field level. Agric. Water Manag..

[11] Park W.B., Moon D.C., Koh G.W. (2012). A study on efficient improvement method of rainwater utilization facilities in Jeju island. J. Soil Groundw. Environ..

[12] Kuller M., Dolman N.J., Vreeburg J.H.G., Spiller M. (2017). Scenario analysis of rainwater harvesting and use on a large scale-assessment of runoff, storage and economic performance for the case study Amsterdam Airport Schiphol. Urban Water J..

[13] Cao X., Ren J., Wu M., Guo X., Wang Z., Wang W. (2018). Effective use rate of generalized water resources assessment and to improve agricultural water use efficiency evaluation index system. Ecol. Indic..

[14] Aljerf L. (2018). Data of thematic analysis of farmer’s use behavior of recycled industrial wastewater. Data Brief..

[15] Mekonnen M.M., Hoekstra A.Y. (2011). The green, blue and grey water footprint of crops and derived crop products. Hydrol. Earth Syst. Sci..

[16] Hoekstra A.Y. Virtual water trade, 12. Proceedings of the International Expert Meeting on Virtual Water Trade.

[17] Hoekstra A.Y., Chapagain A.K., Aldaya M.M., Mekonnen M.M. (2011). The Water Footprint Assessment Manual: Setting the Global Standard.

[18] Zhang Y., Huang K., Yu Y., Yang B. (2017). Mapping of water footprint research: A bibliometric analysis during 2006–2015. J. Clean. Prod..

[19] Novoa V., Ahumada-Rudolph R., Rojas O., Sáez K., de la Barrera F., Arumí J.L. (2019). Understanding agricultural water footprint variability to improve water management in Chile. Sci. Total Environ..

[20] Wu W.P., Zhu Y.F., Zeng W.K., Wang M., Yang D.X., Chen W.F. (2021). Green efficiency of water resources in Northwest China: Spatial-temporal heterogeneity and convergence trends. J. Clean. Prod..

[21] Wang Y.B., Wu P.T., Engel B.A., Sun S.K. (2014). Application of water footprint combined with a unified virtual crop pattern to evaluate crop water productivity in grain production in China. Sci. Total Environ..

[22] Xu Z., Chen X., Wu S.R., Gong M., Du Y., Wang J., Li Y., Liu J. (2019). Spatial-temporal assessment of water footprint, water scarcity and crop water productivity in a major crop production region. J. Clean. Prod..

[23] Cao X.C., Zeng W., Wu M.Y., Li T.Y., Chen S., Wang W.G. (2021). Water resources efficiency assessment in crop production from the perspective of water footprint. J. Clean. Prod..

[24] Zhang S., Tan Q., Zhao H., Zhang T., Zhang T.Y., Hu K.J. (2022). Improving footprint-based water use efficiency through planting structure optimization. Ecol. Eng..

[25] Cao X.C., Zeng W., Wu M.Y., Guo X.P., Wang W.G. (2020). Hybrid analytical framework for regional agricultural water resource utilization and efficiency evaluation. Agric. Water Manag..

[26] Babuna P., Yang X.H., Bian D.H. (2020). Water use inequality and efficiency assessments in the Yangtze river economic delta of China. Water.

[27] Zhang K., Wu F.P., Cheng C.C. (2021). Dynamic Evolution Characteristics of Water Resources Utilization Efficiency in China Under the Constraint of Triple Attribute Carrying Capacity. Environ. Sci..

[28] Shi C.F., Li L.J., Chiu Y.H., Pang Q.H., Zeng X.Y. (2022). Spatial differentiation of agricultural water resource utilization efficiency in the Yangtze River Economic Belt under changing environment. J. Clean. Prod..

[29] Zhang L.L., Ding X.L., Shen Y., Wang Z.Z., Wang X.H. (2019). Spatial Heterogeneity and Influencing Factors of Agricultural Water Use Efficiency in China. Resour. Environ. Yangtze Basin.

[30] Xia L., Shi X.P., Feng S.Y., Qu F.T. (2013). Impact Factor of Farmers’ Water Use Efficiency Under the Background of the Agriculture Enterprise—Minle County in Gansu Province. China Popul. Resour. Environ..

[31] Gong C.Z., Xu C.L., Zhang X.Q. (2020). Spatio-temporal Evolution and Influencing Factors of Water Resources Utilization Efficiency of Cities Along the Middle and Lower Reaches of the Yellow River. Sci. Geogr. Sin..

[32] Han Q., Sun C.Z., Zou W. (2016). Grey water footprint efficiency measure and its driving pattern analysis on provincial scale in China from 1998 to 2012. Resour. Sci..

[33] Su H.W., Liang B.M. (2021). The impact of regional market integration and economic opening up on environmental total factor energy productivity in Chinese provinces. Energy Policy.

[34] Chen Y.B., Yin G.B., Liu K. (2021). Regional differences in the industrial water use efficiency of China: The spatial spillover effect and relevant factors. Resour. Conserv. Recycl..

[35] Song M.L., Wang R., Zeng X.Q. (2018). Water resources utilization efficiency and influence factors under environmental restrictions. J. Clean. Prod..

[36] Cui S.M., Wu M.Y., Huang X., Cao X.C. (2022). Unravelling resources use efficiency and its drivers for water transfer and grain production processes in pumping irrigation system. Sci. Total Environ..

[37] Wang G.F., Chen J.C., Wu F., Li Z.H. (2015). An integrated analysis of agricultural water-use efficiency: A case study in the Heihe River Basin in Northwest China. Phys. Chem. Earth.

[38] Zhang F.T., Xiao Y.D., Gao L., Ma D.L., Su R.Q., Yang Q. (2022). How agricultural water use efficiency varies in China—A spatial-temporal analysis considering unexpected outputs. Agric. Water Manag..

[39] Yang Q., Wu R.W., Wang H.R. (2017). Regional Disparities and Spatial Interaction of Agricultural Water Use Efficiency: 1998~2013. J. Quant. Technol. Econ..

[40] Chapagain A.K., Hoekstra A.Y. (2004). Water Footprints of Nations.

[41] Mekonnen M.M., Hoekstra A.Y. (2010). A global and high-resolution assessment of the green, blue and grey water footprint of wheat. Hydrol. Earth Syst. Sci. Discuss.

[42] Ma H.L., Ding Y.Q., Wang L. (2017). Measurement and Convergence Analysis of Green Water Utilization Efficiency. J. Nat. Resour..

[43] Chukalla A.D., Krol M.S., Hoekstra A.Y. (2018). Grey water footprint reduction in irrigated crop production: Effect of nitrogen application rate, nitrogen form, tillage practice and irrigation strategy. Hydrol. Earth Syst..

[44] Muratoglu A. (2020). Grey water footprint of agricultural production: An assessment based on nitrogen surplus and high-resolution leaching runoff fractions in Turkey. Sci. Total Environ..

[45] Pacetti T., Castelli G., Schröder B., Bresci E., Caporali E. (2021). Water ecosystem services footprint of agricultural production in Central Italy. Sci. Total Environ..

[46] Yang H., Wang X.X., Bin P. (2022). Agriculture carbon-emission reduction and changing factors behind agricultural eco-efficiency growth in China. J. Clean. Prod..

[47] Zhang L., Pang J.X., Chen X.P., Lu Z.M.N. (2019). Carbon emissions, energy consumption and economic growth: Evidence from the agricultural sector of China’s main grain-producing areas. Sci. Total Environ..

[48] Deng X.J., Zhang L. (2022). Spatio-temporal disparity of water use efficiency and its influencing factors in energy production in China. Ecol. Inform..

[49] Chen J., Du M.Z., Huang C.K. (2022). Efficiency and its influencing factors of urban water sector in China and major OECD countries. J. Clean. Prod..

[50] Lv T.G., Liu W.D., Zhang X.M., Yao L. (2021). Spatiotemporal evolution of the green efficiency of industrial water resources and its influencing factors in the Poyang Lake region. Phys. Chem. Earth.

[51] Yu H.Z., Han M. (2017). Spatial-temporal analysis of sustainable water resources utilization in Shandong province based on water footprint. J. Nat. Resour..

[52] Zheng D.F., Hao S., Sun C.Z., Lv L.T. (2018). Spatio-temporal pattern evolution of eco-efficiency and the forecast in mainland of China. Geogr. Res..

[53] Dagum C. (1997). A new approach to the decomposition of the Gini income inequality ratio. Empir. Econ..

[54] Huang B., Wu B., Barry T. (2010). Geographically and temporally weighted regression for spatio-temporal modeling of house prices. Int. J. Geogr. Inf. Sci..

